# Genetic Association and Potential Mediators between Sarcopenia and Coronary Heart Disease: A Bidirectional Two-Sample, Two-Step Mendelian Randomization Study

**DOI:** 10.3390/nu15133013

**Published:** 2023-07-01

**Authors:** Junyi He, Mingkai Huang, Nana Li, Lingfeng Zha, Jing Yuan

**Affiliations:** 1 Department of Cardiology, Union Hospital, Tongji Medical College, Huazhong University of Science and Technology, Wuhan 430022, China; 2 Hubei Key Laboratory of Biological Targeted Therapy, Union Hospital, Tongji Medical College, Huazhong University of Science and Technology, Wuhan 430022, China; 3 Hubei Provincial Engineering Research Center of Immunological Diagnosis and Therapy for Cardiovascular Diseases, Union Hospital, Tongji Medical College, Huazhong University of Science and Technology, Wuhan 430022, China

**Keywords:** Mendelian randomization, sarcopenia, coronary heart disease, cardiometabolic factors, inflammatory biomarkers

## Abstract

Objective: To elucidate the bidirectional correlation of sarcopenia with coronary heart disease (CHD), as well as to investigate the mediating role of cardiometabolic factors and inflammatory biomarkers, a bidirectional two-sample, two-step Mendelian randomization (MR) study was conducted. Methods: Summary statistics were obtained from genome-wide association studies (GWAS). In our bidirectional two-sample MR, genetic variants associated with sarcopenia-related traits and CHD were instrumented for the estimation of bidirectional correlations. Besides, genetic variants associated with thirteen cardiometabolic factors and six inflammatory biomarkers were selected for further mediation analyses. To confirm the consistency of the results, several sensitivity analyses were carried out. Results: Genetically predicted higher appendicular lean mass (OR = 0.835, 95% CI: 0.790–0.882), left hand grip strength (OR = 0.703, 95% CI: 0.569–0.869), right hand grip strength (OR = 0.685, 95% CI: 0.555–0.844), and walking pace (OR = 0.321, 95% CI: 0.191–0.539) reduced CHD risk, while genetic predisposition to CHD did not affect any of the sarcopenia-related traits. Seven mediators were identified for the effects of appendicular lean mass on CHD, including waist-to-hip ratio, hip circumference, systolic blood pressure, low-density lipoprotein cholesterol, total cholesterol, triglycerides, and fasting insulin. The mediation proportion ranged from 10.23% for triglycerides to 35.08% for hip circumference. Hip circumference was found to mediate the relationships between both left (mediation proportion: 24.61%) and right-hand grip strength (24.14%) and CHD, and the link between walking pace and CHD was partially mediated by waist-to-hip ratio (31.15%) and body mass index (26.66%). Conclusion: Our results showed that higher appendicular lean mass, hand grip strength, and walking pace reduced CHD risk, but the causal relationship was not bidirectional. Several mediators were found to mediate the causal pathways between sarcopenia-related traits and CHD, and intervention of these factors may be helpful in terms of CHD prevention in sarcopenia patients.

## 1. Introduction

Cardiovascular diseases (CVD), including coronary heart disease (CHD), continue to have a significant impact on disease burden and mortality rates across the globe [1]. From 1990 to 2019, there was a notable increase in the number of cardiovascular deaths, growing from 12.1 million to 18.6 million, and the incidence of CVD cases exhibited an approximately twofold elevation during this period [2]. Sarcopenia is a degenerative condition of the skeletal muscles that progresses with age and manifests with a concomitant decline in both muscle mass and functional capacity [3]. It is common among the elderly population and influenced by lifestyle, diseases, and genetic factors. The 2019 European Working Group on Sarcopenia in Older People (EWGSOP) stated that the diagnosis of sarcopenia should consider a triad of manifestations, including decreased muscle mass, low muscle strength, and impaired physical performance [4].

There are many observational studies investigating the correlation of sarcopenia with CHD. Campos et al. noted that low muscle mass and walking speed correlate with a high atherosclerotic burden [5]. Decreased muscle mass was identified by Ko et al. as a risk factor for CHD, being independent of other factors [6]. It has also been shown that a decrease in skeletal muscle mass, independent of other cardiometabolic measures, is related to an elevated risk of coronary artery calcification [7]. A study has found that individuals with CHD exhibit a higher prevalence of sarcopenia [8], and the bidirectional relationship of sarcopenia with CVD has also been reported in a recent study [9].

Mendelian randomization (MR) analysis employs genetic variations to evaluate the causality between exposures and outcomes [10]. MR is relatively independent of unmeasured confounders compared to traditional observational studies because the genetic variants were established before disease onset. Several studies employing the MR method have investigated the correlation of sarcopenia-related characteristics with CHD [11,12,13,14,15]. Some have reported a positive correlation between decreased hand grip strength and elevated CHD risk [12], while others have failed to demonstrate this association [13]. Liu et al. reported that reduced HGS, rather than decreased muscle mass, contributed to an elevation in CHD risk [11]. In addition, other MR investigations did not find a correlation between fat-free mass and CHD [14,15], which is not entirely compatible with the observational studies, indicating that the correlation of sarcopenia with CHD remains to be further validated.

CHD is known to be driven by an altered cardiometabolic profile, encompassing elevated systolic blood pressure, increased low-density lipoprotein cholesterol, heightened fasting plasma glucose, and a high body mass index [2]. Associated with aging, immobility, and chronic diseases, sarcopenia can lead to impaired physical activity, insulin resistance, and a persistent inflammatory response, thereby predisposing one to CHD [16]. To our knowledge, the potential mechanisms underlying the correlation of sarcopenia with CHD have not been explored using the MR method. Thus, we conducted a bidirectional two-sample MR study to examine the genetic correlation of sarcopenia-related traits with CHD. Additionally, a two-step MR approach was employed to further explore the probable mediating factors, including cardiometabolic factors and inflammatory biomarkers.

## 2. Materials and Methods

### 2.1. Study Design

MR, a specialized analysis based on genetic instrumental variables (IVs), employs single nucleotide polymorphisms (SNPs) as IVs to evaluate the effects of risk factors on various outcomes, including diseases [10]. SNPs associated with certain risk factors in genome-wide association studies (GWAS) can be utilized as IVs to test their causal effects on different outcomes. The selection of IVs dwelled upon three pivotal assumptions: (1) the IVs must be significantly connected to the exposure; (2) the IVs cannot be connected to any known confounders that could alter the association between an exposure and an outcome; and (3) the IVs must be unrelated to the outcomes and may only affect the outcomes through their effects on the exposure.

**Figure 1:**
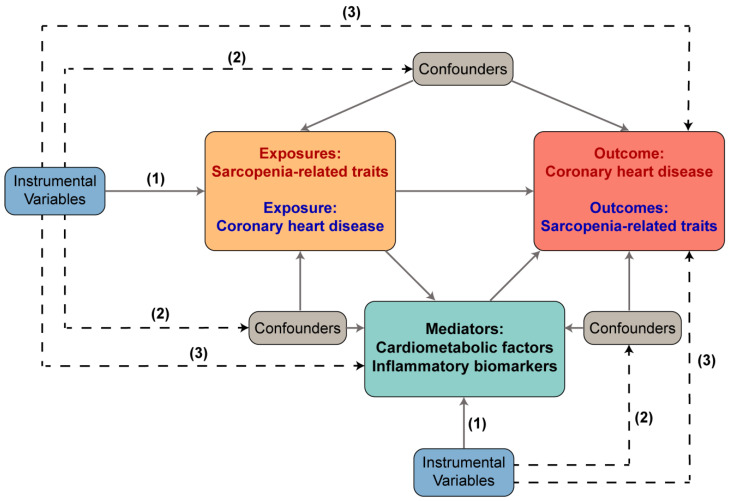
The flowchart of the study based on three assumptions: (1) the IVs must be significantly connected to the exposure; (2) the IVs cannot be connected to any known confounders that could alter the association between an exposure and an outcome; and (3) the IVs must be unrelated to the outcomes and may only affect the outcomes through their effects on the exposure. This Figure illustrates a diagram of our MR study. The dash lines indicate irrelevance, and the solid lines indicate relevance.

Sarcopenia-related traits, including appendicular lean mass (ALM), hand grip strength (HGS), and walking pace (WP), were chosen as indicators of muscle mass, muscle strength, and physical performance, respectively. Using GWAS summary data, a bidirectional two-sample MR method was used to reveal the correlation of sarcopenia-related traits with CHD. To identify potential mediators, a two-step MR analysis was conducted. Specifically, 13 cardiometabolic factors and 6 inflammatory biomarkers were included as potential mediators. We first examined the causal effects of sarcopenia-related traits on each mediator, and then we further assessed the causal effects of possible mediators on CHD. The mediation effects were assessed using the product method [17]. Figure 1 illustrates a diagram of our MR study.

### 2.2. Data Source

ALM was regarded as a pivotal measure of muscle mass in elderly individuals [18]. ALM-related genetic variants were extracted from a GWAS involving 450,243 individuals of European descent [19]. We obtained GWAS summary data for HGS (left, *n* = 461,026; right, *n* = 461,089) and WP (*n* = 459,915) from the UK Biobank cohort. GWAS summary statistics for CHD originated from the CARDIoGRAMplusC4D (Coronary Artery Disease Genome wide Replication and Meta-analysis plus The Coronary Artery Disease Genetics) consortium. It is a meta-analysis comprising 48 GWAS studies, and over 60,000 CHD cases were included [20].

Our investigation included a set of 13 cardiometabolic factors that can be categorized into anthropometric measures, glycemic traits, lipid traits, and blood pressure factors. GWAS summary-level data for anthropometric measures were acquired from the Genetic Investigation of Anthropometric Traits (GIANT) consortium, including body mass index (BMI, *n* = 681,275) [21], waist circumference (WC, *n* = 245,746) [22], hip circumference (HC, *n* = 225,487) [22], and waist-to-hip ratio (WHR, *n* = 224,459) [22]. GWAS data for glycemic traits came from the Meta-Analyses of Glucose and Insulin-related traits Consortium (MAGIC), including fasting insulin (FI, *n* = 151,013), fasting glucose (FG, *n* = 200,622), and hemoglobin A1C (HbA1c) (*n* = 146,806) [23]. GWAS statistics for lipid traits were obtained from the Global Lipids Genetics Consortium (GLGC), including high-density lipoprotein cholesterol (HDL-C, *n* = 187,365), low-density lipoprotein cholesterol (LDL-C, *n* = 173,082), total cholesterol (TC, *n* = 187,365), and triglycerides (TG, *n* = 177,861) [24]. GWAS data for systolic blood pressure (SBP, *n* = 757,601) and diastolic blood pressure (DBP, *n* = 757,601) came from the International Consortium of Blood Pressure [25].

Five common interleukins (ILs), including interleukin 1 receptor antagonist (IL-1Ra), IL-6, IL-8, IL-18, and IL-27, as well as C-reactive protein (CRP), were included in our study as inflammatory biomarkers. Genetic variants for cytokines were from a GWAS study involving 30,931 individuals [26], and summary data for CRP came from a meta-analysis of GWAS studies, encompassing over 200,000 European individuals [27].

### 2.3. Genetic Instrument Selection

The genetic instrument selection followed the following principles: First, SNPs were included if they exhibited a significant association with exposure at the genome-wide level (*p* < 5 × 10^−8^ for sarcopenia-related traits, CHD, and cardiometabolic factors; *p* < 5 × 10^−6^ for inflammatory biomarkers). Second, SNPs that displayed linkage disequilibrium (LD) with an r^2^ greater than or equal to 0.001 were excluded. Third, SNPs that were unavailable for each outcome were excluded, and SNPs that displayed a significant relationship (*p* < 5 × 10^−8^) with the outcome were excluded to avoid violating the aforementioned third assumption. Finally, the F-statistics were computed to assess the potency of each SNP, utilizing the following formula: F = R^2^ (N − K − 1)/(K (1 − R^2^)), in which R^2^ stands for the variance in exposures accounted for by the genetic variance, K represents the number of SNPs, and N represents the sample size. SNPs were chosen based on the commonly cited rule that the F-statistic should be greater than 10 to prevent weak instrument bias in MR analysis [28]. The characteristics of SNPs included in this research are listed in Tables S1–S4.

### 2.4. MR Analysis

A bidirectional two-sample MR analysis was carried out to explore the correlation of sarcopenia-related traits with CHD. A two-step MR analysis was further carried out to further evaluate the probable mediating pathways via 13 cardiometabolic factors and 6 inflammatory biomarkers in the causal relationship.

For primary analyses, we adopted the inverse-variance weighted (IVW) method under the assumption that all genetic variants in the analyses are valid IVs. For sensitivity analyses, first, the weighted median method was chosen, as it provides a reliable estimate of the causal link, assuming that over 50% of the IVs are valid [29]; second, we calculated Cochrane’s Q and Q-derived *p* values [30] to assess the heterogeneity; third, we conducted an MR-Egger regression analysis to reveal possible horizontal pleiotropy by calculating the p valve of its intercept [31]; finally, the MR-PRESSO (MR pleiotropy residual sum and outlier) test was employed, thus correcting for potential confounding factor [32]. In addition, weighted mode and simple mode were adopted to examine the reliability and consistency of the results.

### 2.5. Statistical Analysis

By means of R programming (version 4.1.3), MR analyses were conducted. Two major packages, “TwoSampleMR” and “MRPRESSO”, were used for the estimation of causal effects and the detection of outliers. Results were presented as an odds ratio (OR) along with a 95% confidence interval (CI) per standard deviation. The mediation proportions were calculated according to the formula: (β1 × β2)/β, β stands for the total effect obtained from the primary analysis, β1 stands for the effect of sarcopenia-related traits on mediators, and β2 stands for the effect of mediators on CHD. Standard errors and CIs were calculated using delta methods [33].

## 3. Results

### 3.1. Bidirectional Two-Sample MR Analyses

#### 3.1.1. Causal Effects of Sarcopenia-Related Traits on CHD

**Figure 2:**
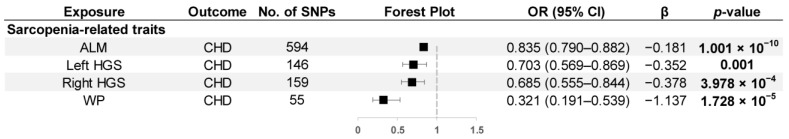
MR estimates derived from the IVW method to assess the causal effect of sarcopenia-related traits on CHD. OR, odds ratio; CI, confidence interval; *p* values < 0.05 are marked bold.

Our findings revealed that genetic variants linked to an elevated ALM (OR = 0.835, 95% CI: 0.790–0.882), left HGS (OR = 0.703, 95% CI: 0.569–0.869), right HGS (OR = 0.685, 95% CI: 0.555–0.844), and WP (OR = 0.321, 95% CI: 0.191–0.539) were correlated with decreased susceptibility to CHD. Figure 2 presents the estimates of the IVW analysis.

#### 3.1.2. Causal Effects of CHD on Sarcopenia-Related Traits

**Figure 3:**
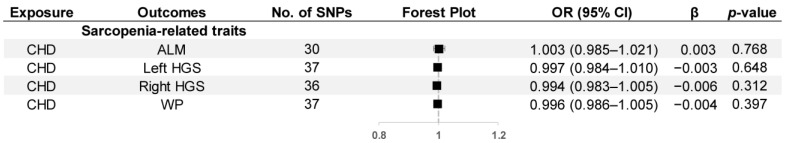
MR estimates derived from the IVW method to assess the causal effect of CHD on sarcopenia-related traits. OR, odds ratio; CI, confidence interval.

The reverse MR analysis found that the genetic predisposition to CHD had no impact on any of the sarcopenia-related traits. Figure 3 displays the estimates of the IVW analysis.

### 3.2. Two-Step MR Analyses

#### 3.2.1. Causal Effects of Sarcopenia-Related Traits on Mediators

**Figure 4:**
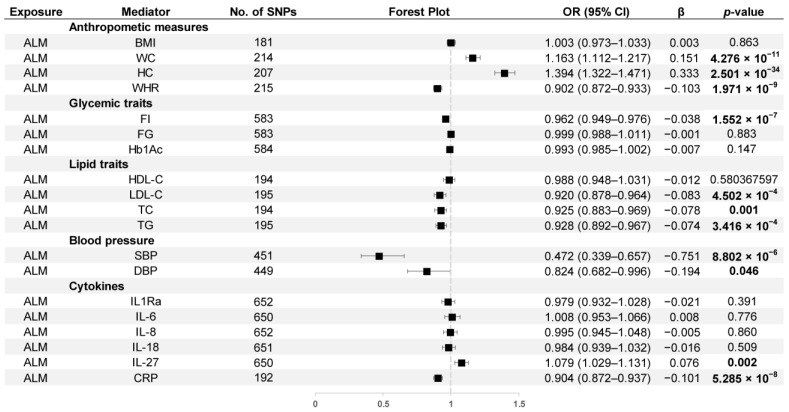
MR estimates derived from the IVW method to assess the causal effect of ALM on thirteen cardiometabolic factors and six inflammatory biomarkers. OR, odds ratio; CI, confidence interval; *p* values < 0.05 are marked bold.

The causal effects of ALM on thirteen cardiometabolic factors and six inflammatory biomarkers using the IVW method are presented in Figure 4. As shown in the figure, ALM was positively related to WC (OR = 1.163, 95% CI: 1.112–1.217), HC (OR = 1.394, 95% CI: 1.322–1.471), and plasma levels of IL-27 (OR = 1.079, 95% CI: 1.029–1.131). Our results also revealed a negative correlation between ALM and several factors, including WHR (OR = 0.902, 95% CI: 0.872–0.933), FI (OR = 0.962, 95% CI: 0.949–0.976), LDL-C (OR = 0.920, 95% CI: 0.878–0.964), TC (OR = 0.925, 95% CI: 0.883–0.969), TG (OR = 0.928, 95% CI: 0.892–0.967), SBP (OR = 0.472, 95% CI: 0.339–0.657), DBP (OR = 0.824, 95% CI: 0.682–0.996), and CRP (OR = 0.904, 95% CI: 0.872–0.937).

**Figure 5:**
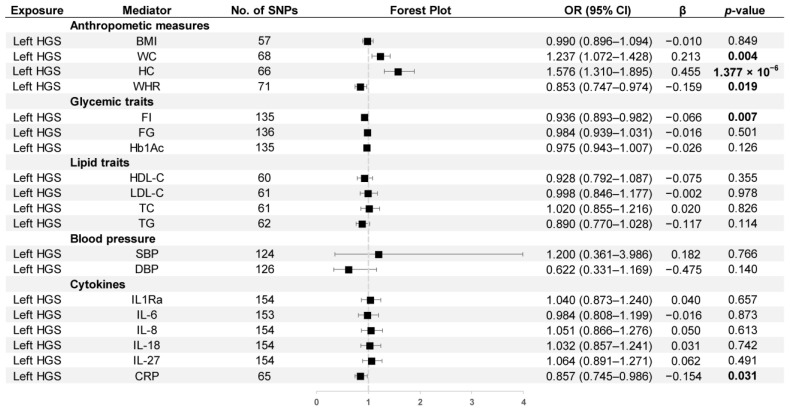
MR estimates derived from the IVW method to assess the causal effect of left-hand grip strength on thirteen cardiometabolic factors and six inflammatory biomarkers. OR, odds ratio; CI, confidence interval; *p* values < 0.05 are marked bold.

Figure 5 depicts the causal effects of left HGS on mediators. Left HGS was positively related to WC (OR = 1.237, 95% CI: 1.072–1.428) and HC (OR = 1.576, 95% CI: 1.310–1.895). The left HGS showed negative correlations with WHR (OR = 0.853, 95% CI: 0.747–0.974), FI (OR = 0.936, 95% CI: 0.893–0.982), and CRP (OR = 0.857, 95% CI: 0.745–0.986).

**Figure 6:**
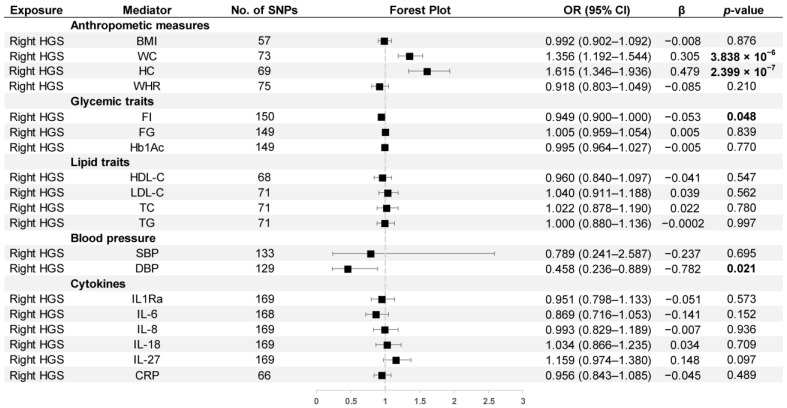
MR estimates derived from the IVW method to assess the causal effect of right-hand grip strength on thirteen cardiometabolic factors and six inflammatory biomarkers. OR, odds ratio; CI, confidence interval; *p* values < 0.05 are marked bold.

Our results found that right HGS was positively associated with WC (OR = 1.356, 95% CI: 1.192–1.544) and HC (OR = 1.615, 95% CI: 1.346–1.936), and it was negatively related to FI (OR = 0.949, 95% CI: 0.900–1.000) and DBP (OR = 0.458, 95% CI: 0.236–0.889). The results are presented in Figure 6.

**Figure 7:**
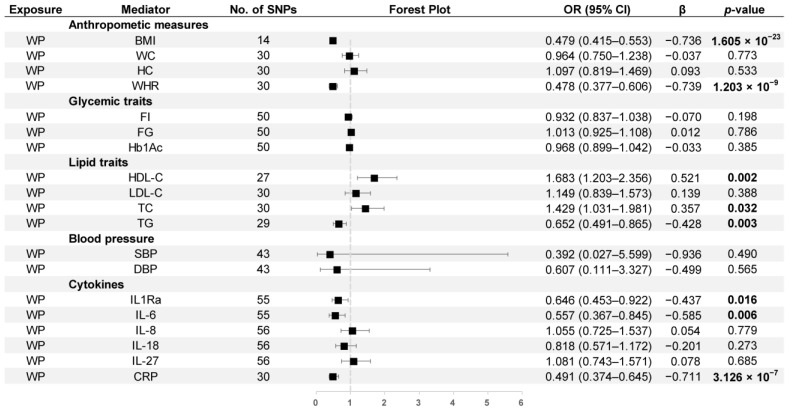
MR estimates derived from the IVW method to assess the causal effect of walking pace on thirteen cardiometabolic factors and six inflammatory biomarkers. OR, odds ratio; CI, confidence interval; *p* values < 0.05 are marked bold.

As illustrated in Figure 7, the positive correlations between WP and HDL-C (OR = 1.683, 95% CI: 1.203–2.356) and TC (OR = 1.429, 95% CI: 1.031–1.981) and the negative correlations between WP and BMI (OR = 0.479, 95% CI: 0.415–0.553), WHR (OR = 0.478, 95% CI: 0.377–0.606), TG (OR = 0.652, 95% CI: 0.491–0.865), IL-1Ra (OR = 0.646, 95% CI: 0.453–0.922), IL-6 (OR = 0.557, 95% CI: 0.367–0.845), and CRP (OR = 0.491, 95% CI: 0.374–0.645) were also observed.

According to the results, nine out of thirteen cardiometabolic factors and four out of six inflammatory biomarkers were chosen as possible mediators, and further MR analysis was conducted to explore the influences of these possible mediators on CHD.

#### 3.2.2. Causal Effects of Possible Mediators on CHD

**Figure 8:**
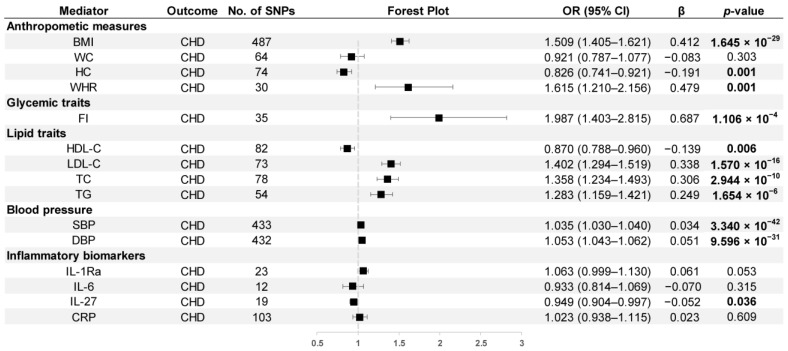
MR estimates derived from the IVW method to assess the causal effect of possible mediators on CHD. OR, odds ratio; CI, confidence interval; *p* values < 0.05 are marked bold.

The estimation of the impact of possible mediators on CHD is shown in Figure 8. Several cardiometabolic factors were observed to be positively correlated with CHD, including BMI (OR = 1.509, 95% CI: 1.405–1.621), WHR (OR = 1.615, 95% CI: 1.210–2.156), FI (OR = 1.987, 95% CI: 1.403–2.815), LDL-C (OR = 1.402, 95% CI: 1.294–1.519), TC (OR = 1.358, 95% CI: 1.234–1.493), TG (OR = 1.283, 95% CI: 1.159–1.421), SBP (OR = 1.035, 95% CI: 1.030–1.040), and DBP (OR = 1.053, 95% CI: 1.043–1.062), while HC (OR = 0.826, 95% CI: 0.741–0.921), HDL-C (OR = 0.870, 95% CI: 0.788–0.960), and IL-27 (OR = 0.949, 95% CI: 0.904–0.997) were negatively correlated with CHD risk.

#### 3.2.3. Mediation Proportion

Based on the results of MR analyses, 11 mediators were selected as potential mediators, of whom the mediation proportions and the 95% CI were calculated. The detailed results are listed in Supplementary Table S13.

Finally, we found eight mediators mediating the correlation of sarcopenia-related traits with CHD. The mediation proportions are listed in Table 1. In the association between ALM and CHD, seven mediators were identified, including HC (mediation proportion: 35.08%), WHR (27.29%), FI (14.53%), LDL-C (15.59%), TC (13.19%), TG (10.23%), and SBP (14.23%). In the relationships of both left and right HGS with CHD, HC (24.61% for left HGS; 24.14% for right HGS) was found to be the mediator. BMI (26.66%) and WHR (31.15%) were considered to be mediators for the impact of WP on CHD.

### 3.3. Sensitivity Analyses

We calculated Cochrane’s Q and Q-derived *p* values to assess the heterogeneity in our estimates. Although significant heterogeneities were found in some of our results, the significance of the IVW estimates remained after adjustment using a random-effects model. Besides, to detect potential pleiotropic effects, we used the p-value corresponding to the MR-Egger intercept. Moreover, we employed the weighted mode, simple mode, MR-Egger, and weighted median to evaluate the impacts of genetically predicted ALM on CVD outcomes, and the results showed relatively high robustness. Tables S9–S12 display the outcomes of our sensitivity analyses. The MR-PRESSO global test was used to resolve heterogeneities, as well as to detect and exclude outliers in our analysis to correct horizontal pleiotropy, and the outcomes after removing these outliers were consistent with the original ones in all of our positive results. The consistency of the sensitivity analyses strengthens the causal reasoning from the primary analyses.

## 4. Discussion

We performed a comprehensive bidirectional two-sample and two-step MR investigation to examine the unclear bidirectional causal association and potential mediators between sarcopenia-related traits and CHD. The results revealed that genetically predicted elevations in ALM, left HGS, right HGS, and WP significantly reduced the CHD risk. The causal relationships were not bidirectional because our results found no evidence that CHD had effects on sarcopenia-related traits. Our two-step MR analysis showed that the excess risk for CHD brought by low ALM was partially mediated by HC, WHR, FI, LDL-C, TC, TG, and SBP, and the most important mediator was HC (35.08%). HC was also found to be a mediator for the causal effects of left and right HGS on CHD, and the causal relationship between WP and CHD was partially mediated by BMI and WHR. Overall, the IVW estimates consistently aligned with those of the sensitivity analyses in terms of both magnitude and direction.

At the observational level, lower muscle mass has been considered an independent CHD risk factor [6]. Additionally, it is reported that increased predicted LBMI (lean body mass divided by height^2^) and ASMI (appendicular skeletal muscle mass divided by height^2^), which represent muscle mass, reduced the risk of CHD in young adults [34]. In our MR results, the genetically predicted ALM was strongly related to CHD, consistent with the aforementioned observational studies. However, previous MR studies failed to establish causality between lean body mass and CHD [11,14,15]. The reason behind this discrepancy in results might be caused by the fact that the body lean mass measurement is likely to be biased by organs, such as the liver and lungs. Besides, ALM has been reported to be more heritable than whole body lean mass [35], giving credibility to our findings. HGS is widely utilized as a proxy for muscular strength [36]. Several MR investigations, focusing on the correlation between HGS and CHD, have drawn different conclusions [11,12,13]. Some found that genetically predicted higher HGS reduced the CHD risk [11,12], while another study suggested no significant correlation between HGS and CHD [13]. The discrepancy in conclusions could be attributed to ethnic variation, as the ancestry of exposure and outcome were not entirely identical. Our study has included more SNPs for the analysis and thus has higher statistical power. Different from the previous studies that investigated the relationship between sarcopenia-related traits, we additionally included walking pace in our analyses. Previous observational studies found that walking pace reduced the CHD risk in men [37], and a slower walking pace has been reported to be strongly correlated to CHD incidence, and this correlation persisted even after accounting for confounders. Zaccardi et al. reported that people with a self-reported slow walking pace had an increased susceptibility to CHD [38]. Consistent with the aforementioned findings, our results suggested that higher gait speed exhibited a strong inverse correlation with genetic susceptibility to CHD.

The mechanism underlying the progression of CHD has been well recognized, and cardiometabolic risk factors, such as elevated SBP, increased FG, elevated LDL-C, and increased BMI, played a detrimental role in it [2]. ALM is predominantly influenced by skeletal muscle mass, and, apart from its contractile function, skeletal muscle also has endocrine functions that affect whole-body metabolism and inflammatory response, so the change in skeletal muscle mass can contribute to several age-related conditions, including myocardial, inflammatory, and metabolic diseases [39,40]. Body-size-adjusted ALM indexes are reported to be indicators for cardiometabolic risk factors, such as lipid profiles, insulin resistance, and blood pressure [41]. Limited studies have examined the intermediary role of cardiometabolic factors and inflammatory biomarkers in the correlation of ALM with CHD. Our study indicates that HC, WHR, FI, LDL-C, TC, TG, and SBP partially contributed to the mediation pathway between ALM and CHD, and, among these mediators, the most important mediators were HC and WHR. “Central obesity”, including WC and WHR, has been reported to be significantly associated with CHD mortality [42]. While gluteofemoral body fat is found to protect against CVD by promoting a beneficial adipokine profile and a decrease in metabolic risks [43], our findings suggested that the management of HC and WHR in sarcopenia patients may have a potential benefit in reducing CHD risk. Clinical studies have observed elevated levels of LDL-C, TC, and TG in sarcopenia patients [44], and people with low skeletal muscle mass index are susceptible to dyslipidemia [45], which is in agreement with our results, as we proved that LDL-C, TC, and TG mediated the relationship between ALM and CHD. Insulin resistance is closely related to risk factors, such as vascular stiffness for diseases of atherosclerotic origin [46,47], and ALM is predominantly influenced by skeletal muscle, and muscular tissue is the predominant tissue responsible for glucose disposal mediated by insulin [48], and our results discovered that FI served as a mediator of the effect of ALM on CHD, suggesting that the increase in ALM may protect against insulin resistance and, therefore, lower the risk of CHD. Being a recognized contributor to CHD [49], the mediation of SBP in the correlation between ALM and CHD has also been observed in our study. According to our results, HC partially mediated the causal effects of left and right HGS on CHD, and BMI and WHR were found to mediate the correlation of WP with CHD. A MR study assessed the potential correlation between adiposity indicators and HGS, and a reverse association between WHR (in males only) and grip strength was observed [50]. According to another MR study, adiposity may play a role in mediating the effect of WP on atrial fibrillation, ischemic stroke, and cardiometabolic stroke [51], indicating that anthropometric measures may act as mediators in the relationship between sarcopenia-related manifestations and CVD, including low HGS and slow WP. Additional evidence from observational studies is needed to confirm this conclusion.

From a mechanistic perspective, the role played by inflammation may contribute to the relationship between sarcopenia and CHD. Inflammation is well recognized to have a crucial role in the progression and initiation of CHD [52,53]. According to previous studies, the levels of pro-inflammatory factors, including IL-6 and CRP, were inversely linked to fat-adjusted ALM, and levels of high-sensitivity CRP (hs-CRP) were found to be negatively correlated with lean mass [54,55]. In addition, the negative relationship between CRP and sarcopenia was confirmed by a meta-analysis [56]. Our investigation did not identify the mediating function of inflammatory biomarkers in the correlation between sarcopenia-related traits and CHD risk, and the reason might be that the roles of cytokines in CHD are sometimes interactive. The changes in serum cytokine levels may not have a direct causal impact on CHD risk, and additional research is necessary to clarify the involvement of different cytokines in mediating the causality between sarcopenia and CHD risk.

Our study comprehensively estimated the genetic relationship between sarcopenia-related traits and CHD, and the results revealed that decreased muscle mass, low muscle strength, and impaired physical performance are risk factors for CHD in sarcopenia patients, suggesting that monitoring muscle mass and strength, along with physical activity, is a critical element in the prevention of CHD among high-risk individuals. In addition, we found that the changes in cardiometabolic factors in sarcopenia patients may increase the risk of CHD, as they were found to mediate the causal pathways between sarcopenia and CHD. These findings suggested that greater emphasis ought to be placed on the levels of these cardiometabolic factors in individuals with sarcopenia, and early interventions with these factors may reduce the incidence of CHD. In a word, our findings provide a theoretical foundation for clinical CHD risk prediction, and monitoring these traits can help lower the prevalence of CHD and thus lower cardiovascular mortality.

Our study has several strengths. Firstly, we proposed a new lens on the causal association and potential mediation between sarcopenia-related traits and CHD using MR. Secondly, by using a bidirectional two-sample MR analysis, the bias caused by reverse causation and confounders, the main limitations of traditional observational studies, was minimized. Thirdly, the statistical power can be increased using summary-level data from GWAS in a two-sample MR approach, especially in assessing effects on binary disease outcomes [57]. Moreover, the SNPs that we selected were significantly related to exposure but not directly related to the outcome. Furthermore, the F-statistic of each SNP was >10 to ensure statistical power. Besides, several sensitivity analyses were conducted to confirm the consistency of causal associations.

Our investigation still has several limitations. First and foremost, although we employed several sensitivity analyses to minimize the bias, the bias would unlikely be eliminated. Secondly, it is worth noting that the GWAS statistics employed in our study were mainly derived from individuals of European descent, which may reduce the impact of ethnic variation, but caution is warranted in applying our conclusions to individuals with different ethnic backgrounds. Thirdly, age and sex are strongly associated with ALM, but we failed to investigate the associations between ALM and CHD in subpopulations because of the lack of individual-level data. Subsequent analyses are warranted to further investigate the correlations stratified by age and sex. Last, although ALM has been chosen as the indicator of muscular mass to avoid the bias caused by non-fat tissue in the measurement of lean mass, and it has been reported that relative muscle mass (ALM/height^2^) is more appropriate when measuring muscle fitness [4].

## 5. Conclusions

In conclusion, this study showed that higher ALM, HGS, and WP were associated with reduced CHD risk, but the causal relationship was not bidirectional. Besides, several cardiometabolic factors were found to mediate the causal pathways between sarcopenia-related traits and CHD, and intervention of these factors may be helpful in terms of CHD prevention in sarcopenia patients.

## Figures and Tables

**Table 1:** The mediation proportion of mediators in the causal relationship between sarcopenia-related traits and CHD.

Mediator	The Effect of Exposure on Outcome β (95% CI)	The Effect of Exposure on Mediator β1 (95% CI)	The Effect of Mediator Outcome β2 (95% CI)	Mediated Proportion (%) (95% CI)
ALM				
HC	−0.181 (−0.236, −0.126)	0.333 (0.279, 0.386)	−0.191 (−0.299, −0.082)	35.08 (11.75, 58.41)
WHR	−0.181 (−0.236, −0.126)	−0.103 (−0.136, −0.069)	0.479 (0.191, 0.768)	27.29 (6.84, 47.74)
FI	−0.181 (−0.236, −0.126)	−0.038 (−0.053, −0.024)	0.687 (0.339, 1.035)	14.53 (4.38, 24.69)
LDL−C	−0.181 (−0.236, −0.126)	−0.083 (−0.130, −0.037)	0.338 (0.257, 0.418)	15.59 (5.01, 26.16)
TC	−0.181 (−0.236, −0.126)	−0.078 (−0.125, −0.031)	0.306 (0.211, 0.401)	13.19 (3.45, 22.93)
TG	−0.181 (−0.236, −0.126)	−0.074 (−0.115, −0.034)	0.249 (0.147, 0.351)	10.23 (2.58, 17.88)
SBP	−0.181 (−0.236, −0.126)	−0.751 (−1.083, −0.420)	0.034 (0.029, 0.039)	14.23 (6.34, 22.11)
Left HGS				
HC	−0.352 (−0.564, −0.140)	0.455 (0.270, 0.639)	−0.191 (−0.299, −0.082)	24.61 (1.90, 47.33)
Right HGS				
HC	−0.378 (−0.588, −0.169)	0.479 (0.297, 0.661)	−0.191 (−0.299, −0.082)	24.14 (2.89, 45.39)
WP				
BMI	−1.137 (−1.656, −0.618)	−0.736 (−0.880, −0.592)	0.412 (0.340, 0.483)	26.66 (12.64, 40.68)
WHR	−1.137 (−1.656, −0.618)	−0.739 (−0.977, −0.500)	0.479 (0.191, 0.768)	31.15 (5.56, 56.73)

Abbreviations: ALM, appendicular lean mass; HGS, hand grip strength; WP, walking pace; BMI, body mass index; HC, hip circumference; WHR, waist-to-hip ratio; FI, fasting insulin; LDL-C, low-density lipoprotein cholesterol; TC, total cholesterol; TG, triglycerides; SBP, systolic blood pressure; CI, confidence interval.

## Data Availability

Publicly available datasets were analyzed in this study. The original contributions presented in the study are included in the article. Further inquiries can be directed to the corresponding author.

## Supplementary Materials

The following supporting information can be downloaded at: https://www.mdpi.com/article/10.3390/nu15133013/s1, Table S1: Characteristics of the genetic instrument variables for the sarcopenia-related traits at the genome-wide significance level (*p* < 5 × 10^−8^); Table S2: Characteristics of the genetic instrument variables for the CHD at the genome-wide significance level (*p* < 5 × 10^−8^); Table S3: Characteristics of the genetic instrument variables for the cardiometabolic factors that are possible mediators at the genome-wide significance level (*p* < 5 × 10^−8^); Table S4: Characteristics of the genetic instrument variables for the inflammatory biomarkers that are possible mediators at the genome-wide significance level (*p* < 5 × 10^−6^); Table S5: Detailed results of MR analysis for causal effects of sarcopenia-related traits on CHD; Table S6: Detailed results of MR analysis for causal effects of CHD on sarcopenia-related traits; Table S7: Detailed results of MR analysis for causal effects of sarcopenia-related traits on mediators; Table S8: Detailed results of MR analysis for causal effects of possible mediators on CHD; Table S9: Detailed results of sensitivity analyses for causal effects of sarcopenia-related traits on CHD; Table S10: Detailed results of sensitivity analyses for causal effects of CHD on Sarcopenia-related traits; Table S11: Detailed results of sensitivity analyses for causal effects of sarcopenia-related traits on mediators; Table S12: Detailed results of sensitivity analyses for causal effects of possible mediators on CHD; Table S13: The detailed mediation proportions of mediators.

## Abbreviations

CHD, coronary heart disease; CVD, cardiovascular disease; MR, Mendelian randomization; GWAS, genome wide association studies; ALM, appendicular lean mass; HGS, hand grip strength; WP, walking pace; IV, instrumental variable; SNP, single nucleotide polymorphism; BMI, body mass index; WC, waist circumference; HC, hip circumference; WHR, waist-to-hip ratio; FI, fasting insulin; FG, fasting glucose; HbA1c, hemoglobin A1C; HDL-C, high-density lipoprotein cholesterol; LDL-C, low-density lipoprotein cholesterol; TC, total cholesterol; TG, triglycerides; SBP, systolic blood pressure; DBP, diastolic blood pressure; IL, interleukin; CRP, C-reactive protein; IVW, inverse-variance weighted; MR-PRESSO, MR pleiotropy residual sum and outlier; OR, odds ratio; CI, confidence interval.
